# 6-(7-nitro-2,1,3-benzoxadiazol-4-ylthio) hexanol: a promising new anticancer compound

**DOI:** 10.1042/BSR20171440

**Published:** 2018-02-13

**Authors:** Huan-huan Sha, Zhen Wang, Shu-chen Dong, Tian-mu Hu, Si-wen Liu, Jun-ying Zhang, Yang Wu, Rong Ma, Jian-zhong Wu, Dan Chen, Ji-feng Feng

**Affiliations:** 1 The Fourth Clinical School of Nanjing Medical University and Nanjing Medical University Affiliated Cancer Hospital, Baiziting42, Nanjing 210009, China; 2 The First Clinical School of Nanjing Medical Universityand Department of Orthopedics, the First Affiliated Hospital of Nanjing Medical University, Guangzhou Road300, Nanjing 210009, China; 3 Department of Biological Science, Purdue University, West Lafayette, IN 47906, U.S.A.; 4 Jiangsu Cancer Hospital and Jiangsu Institute of Cancer Research and Nanjing Medical University Affiliated Cancer Hospital, Baiziting42, Nanjing 210009, China

**Keywords:** anticancer, GSTP1-1 (Glutathione S-transferase Pi), JNK (c-Jun N-Terminal Kinase), NBDHEX (6- (7-nitro-2,1,3-benzoxadiazol-4-ylthio) hexanol)

## Abstract

The 7-nitro-2,1,3-nitrobenzoxadiazole (NBD) derivatives are a series of compounds containing the NBD scaffold that are not glutathione (GSH) peptidomimetics, and result in a strong inhibition of glutathione S-transferases (GSTs). Growing evidences highlight their pivotal roles and outstanding anticancer activity in different tumor models. In particular, 6-(7-nitro-2,1,3-benzoxadiazol-4-ylthio) hexanol (NBDHEX) is extensively studied, which is a very efficient inhibitor of GSTP1-1. It triggers apoptosis in several tumor cell lines and this cytotoxic activity is observed at micro and submicromolar concentrations. Importantly, studies have shown that NBDHEX acts as an anticancer drug by inhibiting GSTs catalytic activity, avoiding inconvenience of the inhibitor extrusion from the cell by specific pumps and disrupting the interaction between the GSTP1-1 and key signaling effectors. Additionally, some researchers also have discovered that NBDHEX can act as late-phase autophagy inhibitor, which opens new opportunities to fully exploit its therapeutic potential. In this review, we summarize the advantages, anticancer mechanisms, and analogs of this compound, which will establish the basis on the usage of NBDHEX in clinical applications in future.

## Introduction

**Figure 1:**
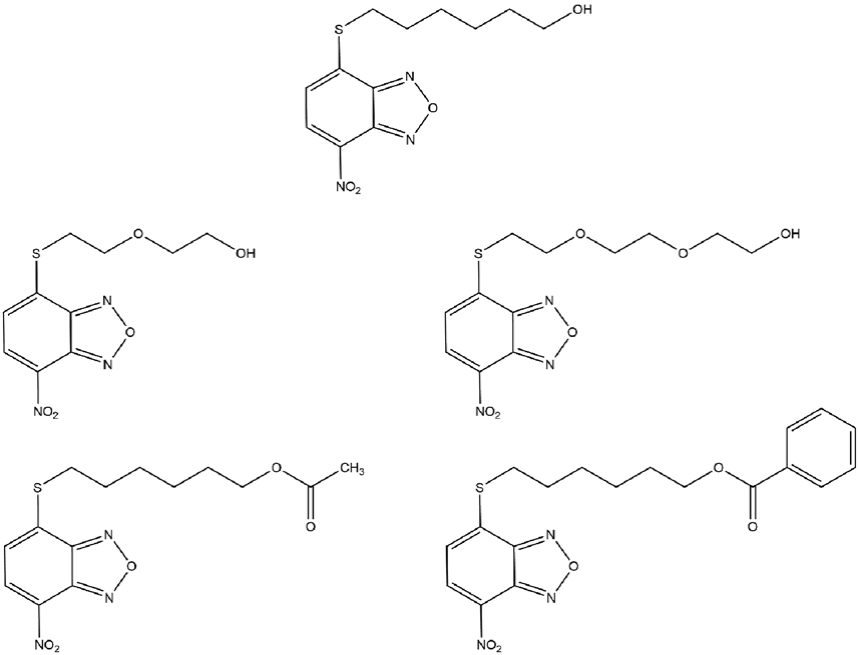
Structures of NBDHEX and its analogs

The 7-nitro-2,1,3-benzoxadiazole (NBD) derivatives are a class of non-glutathione (GSH) peptidomimetic compounds, which are synthesized and characterized as very efficient inhibitors of glutathione S-transferases (GSTs), a family of enzymes involved in xenobiotic detoxification, catalyzing the conjugation of GSH with carcinogens, drugs, toxins, as well as products of oxidative stress [1–5]. The dominant member of GSTs is the GSTP1-1 isoenzyme, that is frequently overexpressed in tumor cells and protects them from apoptosis [1,6,7]. More and more evidence have suggested that NBD derivatives exhibit a remarkable cytotoxicity in several cancer cells at low concentrations, and exert significant therapeutic activity *in vivo* [8–12]. Outstandingly, no treatment-related signs of toxicity are observed in *in vivo* studies on tumor types xenografted in mice [10,11,13]. Amongst the abundant NBD derivatives, 6-(7-nitro-2,1,3-benzoxadiazol-4-ylthio) hexanol (NBDHEX) [2] (Figure 1) has recently emerged as a considerable anticancer compound in multiple malignancies, including leukemia, osteosarcoma, Ewing’s sarcoma, melanoma, rhabdomyosarcoma, mesothelioma, and small cell lung cancer (SCLC), either alone or in combination with antitumor drugs such as cisplatin, doxorubicin, methotrexate, vincristine, and temozolomide [8,10,13–18]. Specifically, NBDHEX inhibits GST’s catalytic activity and is not a substrate of export pumps [9,15,16]. Additionally, it shows activities against cancer cells through disrupting the interaction between the GSTP1-1 and key signaling effectors, which are crucial factors for apoptosis and cell cycle [16,19]. Besides, its ability to weaken the capacity of tumor cells to endure stress conditions via autophagy is recognized [20]. Moreover, *in vivo* studies demonstrate that NBDHEX is effective in reducing both cancer growth and metastatic spread and is well tolerated on various tumor type xenografts in mice [10,13,17]. All these indicate that NBDHEX can display anticancer functions in different cancers.

Here, we will focus on advantages and mechanisms which are associated with the functions of NBDHEX in the cancer progression and treatment. Consequently, given the encouraging results obtained with NBDHEX, we believe that it will become a new potent antitumor agent.

### Advantages of NBDHEX

NBDHEX is a representative molecule of NBD derivatives, a new class of strong and selective inhibitors of GSTs [2]. GST isoenzymes such as GSTP1-1 are overexpressed in many cancer cell lines and can induce drug resistance [6,7,21,22]. Therefore, docking studies aim to design efficient compounds which may modulate their biological activity [2,3,23–25]. However, compounds including ethacrynic acid and GSH derivatives lack class specificity, have scarce affinity, and are often actively extruded from the cell by specific export pumps [3,23,24]. Fortunately, NBDHEX has been shown to overcome these limits, to inhibit GST isoforms at micromolar or submicromolar amounts and to induce cell death in several tumor cell lines [8,10,13]. Although it is not a GSH peptidomimetic compound which has strong specificity of the transferase GSH-binding site (G-site), NBDHEX was conjugated with GSH leading to a stable σ complex in the hydrophobic portion (H-site) of GSTs at the C-4 of the benzoxadiazole ring. H-site is a hydrophobic cavity and as GSTs interact with different hydrophobic toxic species, the H-site normally displays moderate affinity for these compounds [25]. NBDHEX binds efficiently to GSTs and displays lipophilic properties suitable for crossing the plasma membrane [2]. It induces apoptosis and suppresses survival pathways either alone or in combination with conventional anticancer drugs [10,14,15,19] (Table 1). Noteworthy, drug–drug interactions’ analysis considered the combination of NBDHEX with the novel drugs produced synergistic or addictive effects in a lot of cancer cell lines [8,13,18] (Table 2).

**Table 1:** The involvement of NBDHEX in biology process in different cancers

Cancer type	References	*In vitro* or *in vivo*	Effect
Acute T-lymphoblastoid leukemia	[14,15]	*In vitro*	Apoptosis
Chronic myeloid leukemia	[2,14]	*In vitro*	Apoptosis
Acute myeloid leukemia	[9]	*In vitro*	Apoptosis and necrosis
SCLC	[2,16]	*In vitro*	Apoptosis and necrosis
Hepatic carcinoma	[14]	*In vitro*	Apoptosis
Osteosarcoma	[8]	*In vitro*	Apoptosis
	[13]	*In vitro*	Proliferation blockage
		*In vivo*	Against metastatization
	[19]	*In vitro*	Apoptosis and cell cycle arrest
	[20]	*In vitro*	Autophagy inhibition
Ewing’s sarcoma	[13]	*In vitro*	Cell cycle retardation
		*In vivo*	Cytostatic effects
	[11]	*In vitro*	Antiproliferation
		*In vivo*	Tumor growth inhibition
Rhabdomyosarcoma	[13]	*In vitro*	Proliferation blockage
Melanoma	[10]	*In vitro*	Apoptosis and cell cycle arrest
		*In vivo*	Apoptosis and antiproliferation
	[17]	*In vitro*	Apoptosis
		*In vivo*	Tumor growth inhibition
	[12]	*In vitro*	Apoptosis and antiproliferation
Mesothelioma	[18]	*In vitro*	Apoptosis

**Table 2:** Effects of the *in vitro* administration of NBDHEX and conventional anticancer drugs in different cell lines

Cancer type	Reference	Drug	Treatment schedule	Drug–drug interactions
Osteosarcoma	[8]	CDDP	NBDHEX + CDDP	Mostly add
			NBDHEX → CDDP	Add and syn
			CDDP → NBDHEX	Add
	[13]	DX	NBDHEX + DX	Mostly syn
			NBDHEX → DX	Mostly syn
			DX→ NBDHEX	Mostly syn
		MTX	NBDHEX + MTX	Ant
			NBDHEX → MTX	Ant and syn
			MTX→ NBDHEX	Mostly ant
Ewing’s sarcoma	[13]	DX	NBDHEX + DX	Ant
			NBDHEX → DX	Mostly syn
			DX→ NBDHEX	Syn
		VCR	NBDHEX + DX	Add, syn, and ant
			NBDHEX → DX	Mostly syn
			DX→ NBDHEX	Syn
	[11]	ETO	NBDHEX + ETO	Syn
Rhabdomyosarcoma	[13]	DX	NBDHEX + DX	Add
		VCR	NBDHEX + DX	Syn
Melanoma	[17]	TMZ	NBDHEX + TMZ	Syn
Mesothelioma	[18]	CDDP	NBDHEX + CDDP	Syn
			NBDHEX → CDDP	Add and syn

Abbreviations: Add, additive (0.90 ≤ combination index ≤ 1.10); ant, antagonistic (combination index > 1.10); CDDP, cisplatin; DX, doxorubicin; ETO, etoposide; MTX, methotrexate; Syn, synergistic (combination index < 0.90); TMZ, temozolomide; VCR, vincristine.

### Inhibiting GSTs’ catalytic activity

The most widely investigated function of GSTs is the conjugation reaction of several electrophilic compounds, including endogenous and xenobiotic compounds to reduced GSH. Many anticancer drugs are substrates for the GST and thus their conjugation with the GSH can be catalyzed efficiently and extruded from the cell by specific export pumps [26–28]. It is still widely accepted that the detoxifying activity of GSTs exerts a significant part in drug resistance in some tumor cell types via the activation of the GST/GSH cellular system [26,29]. Interestingly, in some cancer cell lines, the enhanced GSTP1 enzymatic activity is very often associated with an increase in *GSTP1* gene expression, increased GSTP1-1 protein level, or both of them. Additionally, the increase in both intracellular levels and enzymatic activity of GSTP1-1 seems to be closely related with the degree of cisplatin resistance [8]. According to these evidences, GSTP1-1 emerges as a potential drug target and NBDHEX acts as strong inhibitors of GST catalytic activity [2,13,17]. However, it needs more evidence to show that NBDHEX can hinder the GST-mediated conjugation of electrophilic anticancer drugs to GSH, and thus may increase intracellular accumulation of the drugs.

### Disrupting the interaction between the GSTP1-1 and key signaling effectors

**Figure 2:**
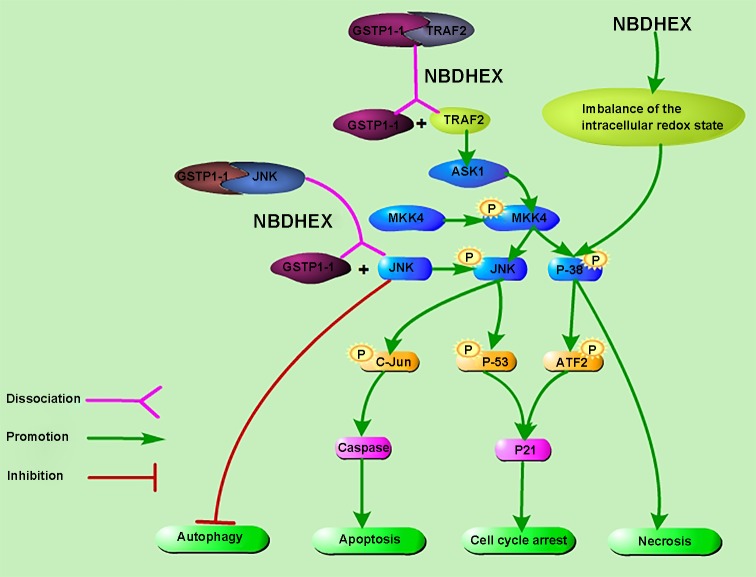
Proposed action mechanism of NBDHEX NBDHEX causes the dissociation of both the GSTP1-1–TRAF2 and the GSTP1-1–JNK complexes and triggers the activation of the MAPK signaling pathway. It induces apoptosis and cell cycle arrest via the phospho-activation of JNK and p38 and their downstream targets including c-Jun, ATF2, and p53. Besides, JNK can participate in impairing autophagy. In addition, the possibility that NBDHEX directly activates p38 through the imbalance of the intracellular redox state cannot be excluded, in which case, cells died by necrosis.

TRAF2 is one of the most ubiquitously expressed TNF receptor-associated factors, a family of proteins interact with a wide range of TNF receptor superfamily members. It directly or indirectly mediates the signal transduction of the receptors involved in the regulation of various cellular responses [30–33]. Of note, it is required for the activation of the apoptosis signal-regulating kinase (ASK1), a mitogen-activated protein kinase kinase kinase (MAP3K). ASK1 can activate both mitogen-activated protein kinase kinase (MKK)4/7–C-jun NH2-terminal kinase (JNK) and MKK3/4/6–p38 signaling pathways [34,35]. De Luca et al. showed experimental evidence that clarified the interaction between GSTP1-1 and TRAF2 and demonstrated the ability of NBDHEX to dissociate the GSTP1-1–TRAF2 complex, further increased the activation of JNK [36]. They also suggested that, in human cisplatin-sensitive and -resistant osteosarcoma cells, GSTP1-1 was able to interfere with the mitogen-activated protein kinase (MAPK) pathway not only at the TRAF2 level, but also at the JNK level [19]. JNK is serine/threonine protein kinase which is at the end of the MAPKs signaling pathway. Cellular processes such as cell growth and apoptosis are closely related to the activation of JNK phosphorylation [37,38]. GSTs can act as JNK regulators through direct association with the JNK, resulting in inhibition of JNK-mediated c-Jun phosphorylation [39,40];

Plenty of studies found that NBDHEX triggered the release of GSTP1-1 from the GSTP1-1–TRAF2 or GSTP1-1–JNK complex, so that it activated the JNK-mediated pathway [15,17,36] (Figure 2). It was reported that in CCRF-CEM and K562 cell lines, the dissociation of the JNK–GSTP1-1 complex was induced by the interaction between NBDHEX and the JNK-linked GSTP1-1and it remained the main pathway of the NBDHEX-triggered apoptosis [14,15]. Also, in melanoma cell lines, NBDHEX activated the JNK pathway through a selective GSTP1-1 targetting and induced apoptosis and cell cycle arrest via the phospho-activation of JNK and p38 and their downstream targets c-Jun, ATF2, and p53 [10,12,17]. Luca et al. [39] showed that GSTP1-1 was unable to inhibit JNK when the active site of the enzyme was occupied by the σ-complex between GSH and NBDHEX. Similar results were obtained in SCLC and mesothelioma cells that NBDHEX activated the JNK signaling pathway [16,18]. Furthermore, NBDHEX promoted a caspase-dependent apoptosis which was unusual in the P-glycoprotein (P-gp) overexpressing cells, and the apoptotic pathway was a direct consequence of dissociation of GSTP1-1from the complex with JNK [15]. It is worth noting that the possibility that NBDHEX directly activated p38 through the imbalance of the intracellular redox state cannot be excluded, in which case, cells died by necrosis (Figure 2). All these results showed that this compound proved to be a promising new strategy by activating vital pathway.

### Overcoming MDR-mediated cancer cell chemoresistance

Multidrug resistance (MDR), a common phenomenon, is a complex clinical problem in oncology and limits the therapeutic effect of anticancer drugs [41,42]. ATP-binding cassette (ABC) efflux pumps including MDR1–P-gp and the MDR protein 1 (MRP1) are one of the well-known MDR mechanisms [43–46]. Both of them are associated with poor treatment outcomes in various kinds of cancers and contribute to the chemoresistance to doxorubicin, vinblastine, etoposide, as well as some other anticancer drugs [47–50]. Unlike most agents, NBDHEX is not a substrate of these export pumps, so that it accumulates in tumor cells and overcomes chemoresistance in cells which are in the presence of ABC transporters conferring the MDR phenotype [9,15,16]. Turella et al. demonstrated that NBDHEX efficiently killed human acute T lymphoblastoid leukemia cell, human osteosarcoma cell, and their selected P-gp variants [15]. Interestingly, their P-gp variants appeared to be more efficiently committed to death by NBDHEX, compared with the parental cell line [15]. Furthermore, they reported that adriamycin-selected, multidrug-resistant human SCLC cell overexpressing MRP1 transporter did not counteract the cytotoxicity of NBDHEX. Actually, NBDHEX induced apoptosis which might be promoted by the very low level of Bcl-2, an antiapoptotic protein found in the multidrug-resistant SCLC cell. Moreover, the decrease in Bcl-2 appeared to be linked to the MDR phenotype [16]. It was also clarified that NBDHEX efflux could not be mediated by either MDR1–P-gp or MRP1 in MDR variants of human acute myeloid leukemia cell [9]. All these findings indicate that NBDHEX may be a very effective compound in killing tumor cells characterized by high levels of MDR1–P-gp or MRP1.

### Inhibiting JNK-mediated last-phase autophagy

The 2016 Nobel Prize in Physiology or Medicine was awarded to the researcher for his discovery of the autophagy machinery [51]. Autophagy is an intracellular catabolic pathway by which cellular macromolecules and dysregulated organelles are engulfed by autophagosome vacuoles, leading to their degradation and breakdown after fusion with autolysosomes [52–55]. Autophagy plays a major role in the progression of established neoplasms by promoting the survival of transformed cells under stress conditions with inadequate supply of oxygen, nutrients, and growth factors [55,56]. Cancer cells live under such microenvironment so that they are predicted to be more susceptible to the suppression of autophagy and tend to activate autophagy constitutively via metabolic reprogramming [54,55,57–60]. Notably, recent investigations indicate constitutive activation of autophagy in tumors is a challenge because it can lead to cancer drug resistance and refractory cancer [61–63]. Such evidences enable us to develop new treatments that inhibit protective autophagy. NBDHEX, acts as autophagy inhibitor, has been reported to impair autophagosome clearance through increasing both LC3-II and the autophagy selective substrate p62. Furthermore, the results were observed in a panel of tumor cell lines of different origins, suggesting that the effects of NBDHEX on autophagy inhibition are general rather than cell type-specific [20]. Interestingly, they also provided evidence that JNK activity was required for autophagy impairment by NBDHEX [20] (Figure 2). In fact, it has been demonstrated previously that suppression of JNK signaling could induce autophagy [64]. By contrast, it could promote autophagy in response to different types of stress signals as well [65,66]. Therefore, improving our understanding of the mechanisms and relationships between NBDHEX, JNK signaling and autophagy may be a significant topic in cancer research.

### Analogs of NBDHEX

However, NBDHEX suffers from relatively low target selectivity because of its high affinity toward GSTM2-2, which is widely expressed in many non-cancerous tissues [2,67]. Also, its poor water solubility limits its oral bioavailability. These findings pointed out the need to search for novel NBDHEX analogs with an improved pharmacological profile. Rotili et al. [68] described a series of 40 NBDHEX analogs bearing phenyl-containing moieties as well as substituted alkyl chains, which replaced the hydroxyhexyl portion at the C4-sulphur atom. Most of the new compounds displayed increased water solubility and higher GSTP1-1 selectivity. Nevertheless, amongst these compounds, some alkyl derivatives possessed cytotoxicity comparable or higher than NBDHEX while the presence of a phenyl ring with polar substituents might result in low cytotoxicity in osteosarcoma U-2OS cells [68]. The lower cytotoxicity might be the consequence of their higher propensity to spontaneously react with the abundant intracellular nucleophile GSH, which was another critical aspect of NBD derivatives. As a further development of their studies, the research team then reported two additional NBDHEX analogs, MC3165 and MC3181 (Figure 1) [69], both of which were designed with the aim of increasing the hydrophilicity and to avoid any significant drop of cytotoxic potency. MC3165 is characterized by bearing one oxygen atom within the hydroxy-containing alkyl chain at the C4 position of the NBD scaffold while MC3181 is characterized by the presence of two. As a result, both the compounds improved hydrophilicity compared with NBDHEX while minimizing the changes into the NBD nucleus and MC3181 indicated more promise in consideration of the water solubility, selectivity toward GSTP1-1, and spontaneous reactivity with GSH [69]. In addition, MC3181 displayed a greater selectivity toward GSTP1-1, high cytotoxicity toward osteosarcoma cells, as well as a panel of different human melanoma cell lines, and exhibited a remarkable therapeutic activity against BRAF-V600E-mutant xenografts [12,20,69]. Similar to NBDHEX, no treatment-related toxicity was observed in xenograft models [12,69]. More recently, two other analogs were designed and synthesized, namely MC2753 and MC2752, the benzoic acid ester and the acetic acid ester of NBDHEX, respectively (Figure 1) [70]. MC2752 did not demonstrate the superiority but it is noteworthy that the presence of a hydrophobic moiety in the side chain strongly affects the mode of interaction between MC2753 with the GSTP1-1, and it did not require GSH to trigger the dissociation of the complex between GSTP1-1 and TRAF2. Therefore, MC2753 would not be affected in its anticancer action by fluctuations of GSH levels [70]. However, MC2753 inhibited only 50% of the enzyme activity and showed lower aqueous solubility compared with NBDHEX, so it may only serve as a lead compound for the development of GSTP1-1 inhibitors not affected by GSH levels. All these results lay the basis for future studies with these analogs of NBDHEX.

## Conclusion and prospects

In this review, we focussed on the pleiotropic roles of NBDHEX in different kinds of cancers. NBDHEX inhibits GSTs catalytic activity, overcomes the MRP1 or P-gp-mediated efflux and disrupts the interaction between the GSTP1-1 and key signaling effectors. Consequently, it induces cell cycle arrest and apoptosis and inhibits autophagy, alone or in combination with novel anticancer drugs. Additionally, NBDHEX analogs are endowed with higher water solubility and increased GSTP1-1 selectivity. Some of them possess cytotoxicity comparable or higher than NBDHEX. Collectively, we suggest that NBDHEX and its analogs may represent a new therapeutic opportunity and open interesting perspectives for cancer therapy in future.

## Abbreviations

ABC: ATP-binding cassette
ASK1: apoptosis signal-regulating kinase
GSH: glutathione
GST: glutathione S-transferase
JNK: C-jun NH2-terminal kinase
MAPK: mitogen-activated protein kinase
MDR: multidrug resistance
MKK: mitogen-activated protein kinase kinase
MRP1: MDR protein 1
NBD: nitrobenzoxadiazole
NBDHEX: 6-(7-nitro-2,1,3-benzoxadiazol-4-ylthio) hexanol
P-gp: P-glycoprotein
SCLC: small cell lung cancer
TRAF2: TNF receptor-associated factor 2
